# Risk assessment and incidence of falls in adult hospitalized patients**^1^**

**DOI:** 10.1590/1518-8345.1551.2862

**Published:** 2017-04-20

**Authors:** Thiana Sebben Pasa, Tânia Solange Bosi De Souza Magnago, Janete De Souza Urbanetto, Mari Angela Meneghetti Baratto, Bruna Xavier Morais, Jéssica Baldissera Carollo

**Affiliations:** 2 MSc, RN, Hospital Universitário de Santa Maria, Santa Maria, RS, Brazil.; 3 PhD, Adjunct Professor, Universidade Federal de Santa Maria, Santa Maria, RS, Brazil; 4 PhD, Adjunct Professor, Pontifícia Universidade Católica do Rio Grande do Sul, Porto Alegre, RS, Brazil; 5 Master's Student, Universidade Federal de Santa Maria, Santa Maria, RS, Brazil.; 6 Master's Student, Universidade Federal de Santa Maria, Santa Maria, RS, Brasil. RN, Hospital Universitário de Santa Maria, Santa Maria, RS, Brazil.

**Keywords:** Nursing, Accidental Falls, Patient Safety, Scales, Hospitalization, Incidence

## Abstract

**Objectives:**

assess the risk of falls in adult hospitalized patients and verify the incidence of the event in this environment.

**Method:**

cohort study, with approval by the Research Ethics Committee, which monitored 831 patients hospitalized at a university hospital. The Morse Fall Scale (MFS) was used to assess the risk and patients with high risk (≥45 points) were considered exposed to falls.

**Results:**

the mean MFS score was 39.4 (±19.4) points. Between the first and the final assessment, the score increased by 4.6%. The first assessment score presented a strong and positive correlation with the final assessment score (r=0.810; p=0.000).

**Conclusion:**

the higher the risk score for falls when the patient is admitted, the higher the score at the end of the hospitalization period and vice-versa. The incidence rate corresponded to 1.68% with a higher percentage of patients classified at high risk of falls.

## Introduction

A fall happens when the individual falls on the floor or moves to levels inferior to the initial position, excluding intentional changes^1^. In hospitalized patients, this incidence figures among the main adverse events the institutions need to prevent^1^. 

Studies appoint the falls as a frequent event in the hospital context, with percentages ranging from 1.1% to 22%, according to the patient's specificity^2^ ^-^ ^3^. This incidence is directly related to patient safety and can increase the length of hospitalization and interfere in the individual's recovery^4^. Falls can be influenced by multiple factors and entail consequences for the patient, such as: damage, extended length of hospitalization and increased care costs^5^.

Assessing the patient and identifying the characteristics that can enhance the probability of falls is fundamental to plan effective prevention strategies^6^. Hence, using specific tools to identify individuals more susceptible to falling can serve as an ally in preventing the incident.

Studies on falls have been undertaken in different scenarios^7^ ^-^ ^9^. In Brazil, however, there is a lack of studies that investigate the incidence of this event in the hospital context and assess the risk by means of validated instruments. In this study, the Morse Fall Scale was used because it is a global tool that permits the effective identification of fall risks in hospitalized adults. The tool was also chosen because it has been translated and cross-culturally adapted to Portuguese^10^. In this context, the goal was to assess the risk of falls in adult hospitalized patients and to verify the incidence of the event in this environment.

## Method

Cohort study developed at the Surgical Clinical and Medical Clinical services I and II of a teaching hospital located in the interior of the State of Rio Grande do Sul, Brazil. The study was developed between March and July 2013 and includes all patients hospitalized at the proposed services; over 18 years of age and who accepted to participate in the research. The ideal time to start the collection was set as up to 24 hours of hospitalization. To minimize the losses, however, this time was expanded to up to 48 hours. No exclusion criteria were established.

The data collection started after the project had been approved by the Research Ethics Committee at Universidade Federal de Santa Maria - CEP/UFSM, under opinion 206.995, on February 25^th^ 2013. The patients were included after the patient or the companion had signed the Informed Consent Form.

To collect the data, information from the patient history was assessed: age, sex, date of hospitalization and discharge, medical diagnoses and registers of falls. In addition, the patient was assessed for: muscle strength in upper and lower limbs^11^, *Morse Fall Scale (MFS*)^10^ score and occurrence of falls. It is highlighted that the patient was monitored across the hospitalization period and that collaborators the researcher had trained in advance collected the data daily.

The data were organized in *Excel* ^(r)^, version 2010, with independent double data entry. After checking for errors and inconsistencies, the analysis was developed in the *software Predictive Analytics SoftWare* (PASW, SPSS, USA, 2011) version 18.0 *for Windows*.

The descriptive statistical analysis of the results was undertaken by means of absolute and relative frequencies for the categorical variables; and means, standard deviations and medians for the continuous variables, according to the symmetry of the data. The Kolmogorov-Smirnov test was applied to investigate the normality distribution of the continuous variables. For the comparison between two independent groups of the continuous variables, Student's t-test (symmetrical distribution) and Mann Whitney's test (asymmetrical distribution) were used; to compare the categorical variables, Pearson's Chi-squared or Fisher's Exact test were used. To investigate the linear relation between the MFS scores on the first and final assessment, Pearson's Correlation was applied. For statistical decision criteria, in all comparisons, statistical significance (α) was set at 5%.

The incidence rate (IR) was calculated as the ratio between the number of new cases of falls and the total of person-time produced between on the total number of patients monitored, according to the equation^12^: *TI _(t0 - t)_ = I / PT*, where *(t_0_ - t)* refers to the interval between the baseline *t_0_* and moment t; *I* represents the number of new cases that emerged between t_0_ and t; and *PT* represents the quantity of person-time the population accumulated during the study.

The decrease in the muscle strength can be a factor predisposing to falls and is not included in the MFS. Thus, the test by Rossi and Mistrorigo^11^, scored from zero to five, was used to assess the muscle strength in each upper and lower limb. The higher the score, the greater the patient's muscle strength. For the analyses, the limb assessment was grouped in upper and lower limbs and the score was dichotomized into reduced (0 to 4 points) and preserved (5 points).

The MFS consists of six items with mutually different scores, which are attributed to each patient and can range between 0 and 125 points. Patients classified between 0 and 24 points are at low risk of falls during the hospitalization; patients classified between 25 and 44 points are at moderate risk of falls; and patients with 45 points or more are at high risk of falls^10^. Patients classified as high risk were considered exposed to falls (MFS score of 45 or higher). Low and moderate-risk patients (MFS between 0 and 44) were considered not exposed to the event.

## Results

Among the 864 patients hospitalized at the investigated services between March 11^th^ and July 11^th^ 2013 who complied with the inclusion criteria, 831 were monitored daily to assess the risk and occurrence of falls. The losses (N=33; 3.8%) were due to refusals to participate.

In this study, male patients were predominant (N=500; 60.2%), between 67 and 92 years of age (N=284; 34.2%), with a mean age of 58.1 (±16.1) years. The mean length of hospitalization was 7.7 days (±9.2), median 4 days. On average, the patients were monitored for 5.4 days (±5.2), with a median of 4 days (minimum 1 and maximum 27 days).

In Table 1, the descriptive statistics are displayed for the Morse Fall Scale *(MFS)* according to the length of hospitalization.

**Table 1:** Descriptive statistics for Morse Fall Scale score according to length of hospitalization. Santa Maria, RS, Brazil, 2013 (N=831)

Morse Fall Scale (MFS)	N	Minimum Score	Maximum Score	Mean	Standard Deviation
General Average*	831	0	110.0	39.4	19.4
Standard Deviation	661	0	33.44	5.3	6.4
Variation Coefficient	649	0	0.185	0.177	0.296

*Referring to 122 days of monitoring.

The patients' mean score was 39.4 points, with a minimum of 0 and maximum of 110. The standard deviation of the MFS, that is, the internal variation for a same patient during the period, was about 5.3 points, far below the minimum MFS score of 15 points. That indicates a homogeneous score in the course of the hospitalization.

The Variation Coefficient of the MFS is similar to its standard deviation, but related to the patient's average score. Hence, one may say that, on average, the same patient's score during the period assessed varied by 18.5%. It is highlighted that, in total, 337 patients presented zero variation in the MFS score during the period (one assessment day or MFS score equal to zero). 

In Table 2, the patients' distribution according to the MFS items is described. 

**Table 2:** Patient distribution according to items of the Morse Fall Scale (MFS) during the monitoring period (11/03 till 11/07). Santa Maria, RS, Brazil, 2013

Item Morse Fall Scale (MFS)	N	%
History of falls		
Yes	203	24.4
No	628	75.6
Secondary Diagnosis		
Not more than one medical diagnosis	325	39.1
More than one medical diagnosis	506	60.9
Use of intravenous device		
Yes	771	92.8
No	60	7.2
Help with walking		
None; Totally bedridden; Help by Health Professional	710	85.4
Uses Crutches/Cane/Walker	53	6.4
Holds on to Furniture/Wall	68	8.2
Walking		
Normal; Does not walk/ Totally bedridden/ Uses Wheelchair	411	49.5
Weak	258	31.0
Committed/ staggering	162	19.5
Mental Status		
Oriented in terms of capacity/limitation	760	91.5
Overestimates capacity/ Forgets limitations	71	8.5

In the fall history, 24.4% (N=203) of the patients presented a score different from zero (25 points) on at least one of the days investigated, while 75.6% (N=628) scored zero on all days investigated. For the secondary diagnosis, 39.1% (N=325) of the patients did not present more than one medical diagnosis during the 30 days investigated. The other patients investigated (N=506; 60.9%) scored higher than 15 points, that is, more than one medical diagnosis.

As regards the use of an intravenous device, the results appointed that 92.8% (n=771) presented this characteristic on at least one of the 30 days investigated. Concerning help with walking, 85.5% (N=710) did not need any kind of help; 6.4% (N=53) needed crutches, a cane or walker; and 8.2% (N=68) did not use any type of help with walking but held on to the furniture or wall on at least one of the 30 days investigated.

What walking is concerned, 49.5% (N=411) of the patients presented score zero only (normal walking; does not walk/ Totally bedridden/ Uses wheelchair) on the 30 days investigated; 31% (N=258) scored 10 (weak walking) on at least one of the 30 days investigated; and 19.5% (N=162) of the patients scored 20 (committed or staggering walk) on at least of the 30 days assessed. Concerning the mental status, 91.5% (N=760) of the participants were oriented in terms of the capacity/limitation to walk alone, that is, they only scored zero on the 30 days assessed.

In Table 3, the patients' risk classifications for falls on the first and final assessment day and the mean classification are described, according to the MFS score.

**Table 3:** Distribution of patients according to risk classification on the Morse Fall Scale (MFS) on the first, final and mean assessments. Santa Maria, RS, Brazil, 2013 (N=831)

Classification of risk for falls - Morse Fall Scale (MFS)	N	%
Morse Fall Scale (MFS) - First Assessment		
Low	255	30.7
Moderate	272	32.7
High	304	36.6
Morse Fall Scale (MFS) - Final Assessment		
Low	212	25.5
Moderate	277	33.3
High	342	41.2
Morse Fall Scale (MFS)- Mean assessments		
Low	210	25.6
Moderate	308	37.1
High	313	37.7

When the patients' risk of falls was assessed, according to the MFS classification, on the first as well as the final assessment and on average, the highest percentage of patients was classified in the category high risk for falls (36.6%, 41.2% and 37.7%, respectively). Between the first and final assessments, the MFS score increased by 4.6%. The score on the first assessment revealed a strong and positive correlation with the score on the final assessment (r=0.810; p=0.000), that is, the higher the risk score for falls when the patient was admitted, the higher the score at the end of the hospitalization period and vice-versa.

During the 122 days of monitoring, among the 831 patients assessed, 19 dropped to the floor. That implies an average 4.7 falls per month. The fall incidence rate per person/day in the total group of 6400 patients/day corresponded to 1.68% (95%CI; 1.51 - 1.72%). As regards the accumulated frequency, which directly estimates the probability/risk that an individual develops the outcome during a specific time period, was equal to 2.28 (95%CI: 1.66 - 2.91).

In Table 4, the absolute and relative frequencies of the patients with and without falls are displayed, according to demographic variables, health conditions and MFS classifications.

**Table 4:** Distribution of patients according to demographic variables, health conditions and classifications on the Morse Fall Scale (MFS). Santa Maria, RS, Brazil, 2013 (N=831)

Variables	Fall					p
	No			Yes		
	N	%		N	%	
Sex						0.838*
Female	323	97.6		8	2.4	
Male	489	97.8		11	2.2	
Age						0.609*
18 to 59 years	390	98.0		8	2.0	
60 to 92 years	422	97.5		11	2.5	
Physical exercise						0.183**^†^**
No	603	97.3		17	2.7	
Yes	209	99.1		2	0.9	
Musculoskeletal Problem						0.840*
No	531	97.8		12	2.2	
Yes	281	97.6		7	2.4	
Visual Difficulty						0.701*
No	224	97.4		6	2.6	
Yes	588	97.8		13	2.2	
Hearing Difficulty						0.009†
No	676	98.4		11	1.6	
Yes	136	94.4		8	5.6	
Muscle Strength						
Upper Limbs						0.891*
Reduced	589	97.8		13	2.2	
Preserved	223	97.4		6	2.6	
Lower Limbs						0.262*
Reduced	587	98.2		11	1.8	
Preserved	225	96.6		8	3.4	
Morse Fall Scale - Mean Assessments						<0.001**^‡^**
Low Risk	210	100.0		--	---	
Moderate Risk	307	99.7		1	0.3	
High Risk	295	94.2		18	5.8	
Morse Fall Scale - First Assessment						<0.001**^‡^**
Low Risk	254	94.6		1	0.4	
Moderate Risk	270	99.3		2	0.7	
High Risk	288	94.7		16	5.3	
Morse Fall Scale - Final Assessment						<0.001**^‡^**
Low Risk	212	100.0		--	--	
Moderate Risk	276	99.6		1	0.4	
High Risk	324	94.7		18	5.3	

Pearson's Chi-squared test with continuity correction; † Fisher's Exact Test;

‡Chi-squared test with Monte Carlo correction.

The patients with hearing problems presented a significantly higher percentage of falls (N=5; 5.6%) when compared to the patients without this difficulty. As regards the risk classification according to the MFS, falls victims obtained a significantly higher percentage in the high risk category (p<0.001). 

**Figure 1:**
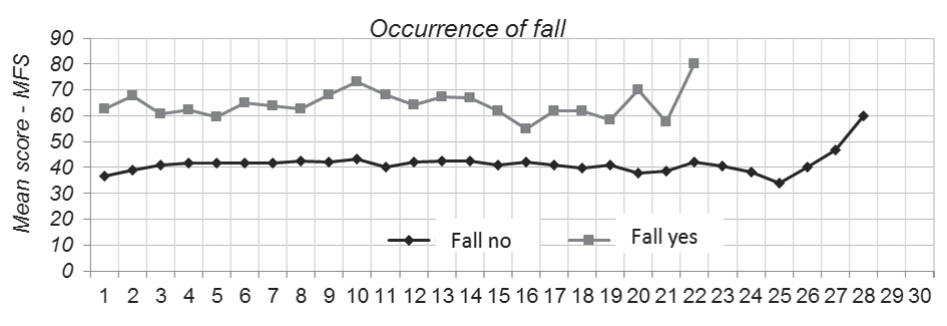
Mean MFS score on each day assessed for the presence and absence of falls.

When comparing the MFS scores with the presence and absence of falls between the groups (with and without falls), a higher MFS score was detected across the assessment period in the group with falls (Figure 1).

When the scores were compared intragroup, it was observed that, among the patients who did not present falls, the average scores ranged between 33.8 and 60.0 points on the MFS. In the group with falls, however, the mean scores ranged between 55.0 and 80.0 points, that is, a higher variation when compared to the group without falls.

## Discussion

What the length of the assessment and, inherently, of the hospitalization was concerned, most patients were assessed between two and ten times, with an average length of hospitalization of 7.7 days (±9.2). In that sense, in another study, on average, the assessed patients were in hospital for 3.1 days (±2.57) and the length of hospitalization was longer in cases of falls^13^. Hence, the longer the length of hospitalization, the greater the patient's risk of falls (OR=3.2; *p*<0.01)^13^.

As regards the mean MFS scores, previous studies found averages that differed from this study (39.4 ±19.4 points). In a study that monitored patients similar to the persons assessed in this study, the mean MFS score was 31.7 (±16.9), corresponding to a moderate risk of falls^13^. In another study, a higher mean MFS score was found (57.2), corresponding to a high risk of falls^7^. The latter was developed at a rehabilitation service, where a higher percentage of patients experienced limitations and difficulties, mainly related to walking^7^. Hence, the mean MFS scores and, consequently, the profile of the hospitalized patients will depend on the service offered in hospital.

In this study, a higher percentage of patients obtained scores indicating risk of falls on the MFS items: secondary diagnosis and use of intravenous device. In one study^14^, a similar result was found, in which the patients assessed presented a higher prevalence associated with the risk of falls only for the item use of intravenous device (83.3%). In another study, 40.7% of the patients assessed scored for the Nursing Diagnosis (ND) and risk for falls on the item secondary diagnosis, that is, they received more than one diagnosis^15^. These two items are important, as well as their relation with medication use, leading to the need for falls prevention strategies related to the use of medication.

It is important to highlight the item walking. When adding up the percentages of patients with weak and or compromised/staggering walking, 50.5% of the patients monitored revealed some walking problem on at least one assessment day. In that sense, the health professionals should assess the patients in terms of autonomy and the need to use walking accessories^16^. Another important strategy is to advise the patients and companions to turn them into partners in care as, when they are able to perceive their limitations in terms of impaired mobility, it becomes easier to request help.

The patients' classification according to the MFS, on the first and final assessments and on average, appointed that a higher percentage was classified at high risk for falls, and was therefore classified as exposed to the event. That is in accordance with a Brazilian study that used the MFS to assess hospitalized patients, showing a high risk of falls^14^.

What the MFS classification is concerned, a previous study observed a significant but mild drop in the scale scores when the first and final assessments were compared (57.2 vs. 51.6)^7^. This evidence differs from this study, in which, the higher the MFS score on the first assessment, the higher the score on the final assessment, with statistical significance. This finding strengthens the need to assess the patient when admitted to the service, with periodical reassessments. In that sense, the assessment should be done daily, enhancing the reassessments in case of transfer from the sector, identification of another risk factor, change of clinical conditions and occurrence of falls^16^. Through this monitoring, changes in the scores and risk factors can be identified and the strategies can be remodeled when necessary.

Concerning the incidence rate of falls, the percentages show some variation between the studies. Research developed at inpatient services including patients similar to the persons monitored in this study appointed falls incidence rates of 1.8% and 2.1%^2^ ^,^ ^8^. These authors highlight the lower rates after the implementation of preventive strategies (1.1% and 1.5%). The comparisons demonstrate that the incidence rate and the percentage of falls in this study are in accordance with the percentages found in the Brazilian and international literature (1.3% to 12.6%)^17^ ^-^ ^19^.

In the analysis of the research variables, when comparing demographic data, physical exercise, health conditions and MFS classification between patients with and without falls, only the variable hearing problem was significantly higher among fall victims. No other studies were found that supported the findings, alerting to the need to better investigate the association between hearing problems and the occurrence of falls. Authors^20^ investigated hearing impairment as a factor predisposing to falls but found no significant result. 

What the other findings are concerned, other studies did not evidence a significant difference either for falls related to sex^7^ ^,^ ^13^ and age^7^. Regarding the variable musculoskeletal problem, these study results differed from the findings in other studies^14^ ^,^ ^18^ that found a significant association between high risk for falls and the presence of musculoskeletal disorders.

The association between the degree of risk based on the MFS scores and the presence or not of falls was significant. On average, a larger percentage of falls victims were classified as at high risk for falls (≥45 points). In that sense, the MFS score of the falls victims was relatively higher when compared to patients who did not fall (65.1 vs. 55.2)^21^.

Thus, using this tool to classify the patients and, based on the risk identification, listing prevention strategies, turns into an ally in the nurse's work process and in the promotion of patient safety in the hospital context.

Based on the results, some strategies can be cited that can be included in the care plan: use specific instruments to predict the risk of falls, one of which is the MFS: train the team on the appropriate way to assess the patient and implement the strategies; advise patients/companions on the risk factors that can entail falls; and identify high-risk patients, using a signal at the headrest or a specific wristband, among other strategies^7^ ^-^ ^8^ ^,^ ^22^.

The assessment period is appointed as a limitation, considering that the prevalence of the investigated outcome is low, demanding a larger number of participants in the research. Greater investments in longitudinal studies are needed at Brazilian institutions due to the multifactorial nature of falls. This study contributes to the knowledge, appointing the incidence of falls in adult patients and the importance of using a globally validated tool for the purpose of risk assessment.

## Conclusion

The largest group of hospitalized patients was classified as at high risk for falls according to the MFS. The incidence rate of falls corresponded to 1.68% and it was verified a higher percentage of patients who fell were classified in the category high risk for falls. These data signal that the MFS can be used to assess the risk of falls, with a view to identifying factors that contribute to the occurrence of this incident in the hospital context, as the scale assesses different items.

Although low, the incidence rate of falls detected in this study appoints the need to sensitize the health professionals to the occurrence of these incidents in hospitals. Being closer to the patient, the nursing team is an important ally in the prevention of falls. This proximity permits the early identification of risk situations and favors the nurse's planning of actions, in cooperation with the multidisciplinary team, with a view to reducing the falls rate, which interferes in the continuity of care and in patient safety.
